# The Association Between the Triglyceride–Glucose Index, Its Combination with the Body Roundness Index, and Chronic Kidney Disease in Patients with Type 2 Diabetes in Eastern China: A Preliminary Study

**DOI:** 10.3390/nu17030492

**Published:** 2025-01-29

**Authors:** Xiangyu Chen, Xiaofu Du, Feng Lu, Jie Zhang, Chunxiao Xu, Mingbin Liang, Lijin Chen, Jieming Zhong

**Affiliations:** Department of Non-Communicable Disease Control and Prevention, Zhejiang Provincial Center for Disease Control and Prevention, Hangzhou 310051, China; xychen@cdc.zj.cn (X.C.); xfdu@cdc.zj.cn (X.D.); flu@cdc.zj.cn (F.L.); jiezhang@cdc.zj.cn (J.Z.); chxxu@cdc.zj.cn (C.X.); mbliang@cdc.zj.cn (M.L.); ljch@cdc.zj.cn (L.C.)

**Keywords:** chronic kidney disease, triglyceride–glucose index, body roundness index, type 2 diabetes

## Abstract

Objectives: This study aimed to investigate the relationship between the triglyceride–glucose (TyG) index, its combination with the body roundness index (BRI), and chronic kidney disease (CKD) in patients with type 2 diabetes mellitus (T2DM) based on a cross-sectional analysis conducted in Eastern China. Methods: The research originates from a cross-sectional study performed in Zhejiang Province, Eastern China, between March and November 2018. The TyG-BRI index was calculated based on triglyceride, fasting blood glucose, and the BRI. The correlation between the TyG-BRI index and the risk of CKD was assessed using a restricted cubic spline model. A multivariate logistic regression model was used to investigate the association between the TyG-BRI index and the risk of CKD. Receiver operating characteristic (ROC) analyses was used to evaluate the optimal cut-off and value of the TyG-BRI index for predicting CKD. Results: A total of 1756 T2DM participants were enrolled in this study. The TyG-BRI index was significantly higher in participants with CKD than in those without CKD. In the fully adjusted model, the odds ratios for CKD in the second, third, and fourth TyG-BRI quartiles were 0.93 (95% CI: 0.65–1.33), 1.33 (95% CI: 0.94–1.88), and 1.57 (95% CI: 1.10–2.25), respectively, compared to the first quartile. RCS analysis confirmed a linear dose–response relationship between the TyG-BRI index and CKD risk (all *p* for nonlinearity > 0.05). ROC curve analysis revealed that the TyG-BRI index had moderate predictive value for CKD, with an area under the curve of 0.626 (95% CI: 0.597–0.656, *p* < 0.001). The optimal cut-off value for the TyG-BRI index was 42.46, with a sensitivity of 68.2% and specificity of 52.2%. Conclusions: The TyG-BRI index was positively associated with the risk of CKD in a T2DM population, demonstrating a dose–response relationship and moderate predictive value. It may serve as a valuable tool for identifying high-risk individuals and informing targeted interventions to prevent or delay CKD progression in this population.

## 1. Introduction

The worldwide incidence of type 2 diabetes mellitus (T2DM) is escalating at a concerning pace, posing great challenges to global health systems and societal progress [1]. This issue is particularly pronounced in China, where the number of T2DM cases has been steadily rising [2]. As T2DM becomes more prevalent, the occurrence of associated complications, especially chronic kidney disease (CKD), is also on the rise [3]. CKD represents a frequent and serious comorbidity in individuals with T2DM, significantly contributing to mortality and healthcare expenditures [3]. The simultaneous presence of T2DM and CKD exacerbates the clinical outcomes for both conditions, underscoring the critical importance of identifying modifiable risk factors to mitigate the impact of CKD in this high-risk group.

A key factor in the pathogenesis of T2DM is insulin resistance (IR), which also plays a critical role in the development of CKD [4,5,6]. IR is characterized by the impaired ability of the body to utilize insulin effectively, resulting in hyperglycemia and subsequent metabolic dysregulation [7]. These metabolic disturbances contribute to CKD progression through mechanisms such as glomerular hyperfiltration, increased renal inflammation, and fibrosis [8,9]. Beyond its central role in T2DM and CKD, IR is also a defining feature of polycystic ovary syndrome (PCOS), a common endocrine disorder in women of reproductive age [10]. PCOS is associated with significant cardiometabolic risks, including impaired glucose tolerance, dyslipidemia, and hypertension, which are mediated in part by IR [11]. The shared pathophysiological role of IR across these conditions underscores its systemic impact and highlights the importance of identifying reliable and accessible markers of IR. In the context of T2DM, such markers are crucial for early detection and effective risk stratification of CKD, ultimately improving clinical outcomes.

Current approaches for evaluating IR are frequently intricate, invasive, and expensive, making them less practical for use in primary care environments [12]. In contrast, the triglyceride–glucose (TyG) index, calculated using fasting triglyceride and glucose concentrations, offers a more straightforward and economical alternative for assessing IR [13]. This index captures metabolic changes linked to IR and has demonstrated potential as a reliable proxy measure. Moreover, obesity-related indicators, such as the body roundness index (BRI), aggravate IR and play a significant role in the progression of CKD. Nevertheless, the combined influence of the TyG index and obesity-related metrics on CKD risk, especially among individuals with T2DM, has not been thoroughly investigated.

This study aims to explore the relationship between the TyG-BRI index and the risk of CKD in T2DM patients in Eastern China, a region facing a rapid rise in both T2DM and CKD prevalence. The novelty of this study lies in its integration of two easily measurable, cost-effective indices—the TyG index and BRI—to assess CKD risk in an underserved and rapidly expanding population. By combining these indices, this study seeks to provide valuable insights into more accessible and practical methods for identifying individuals at high risk of CKD. Ultimately, the goal is to contribute to the development of targeted, preventive strategies to delay or mitigate CKD onset, addressing a significant public health challenge in this region.

## 2. Materials and Methods

### 2.1. Study Population

This cross-sectional investigation, carried out between March and November 2018, formed a segment of the China National Diabetic Complications Study [14]. Detailed information on this study’s design, methodology, etc., is available in a previous report [14]. To summarize, the study included participants drawn from the local diabetic resident population. Eligible individuals were required to be at least 18 years old, possess cognitive ability, not be pregnant, and not be bedridden.

**Figure 1:**
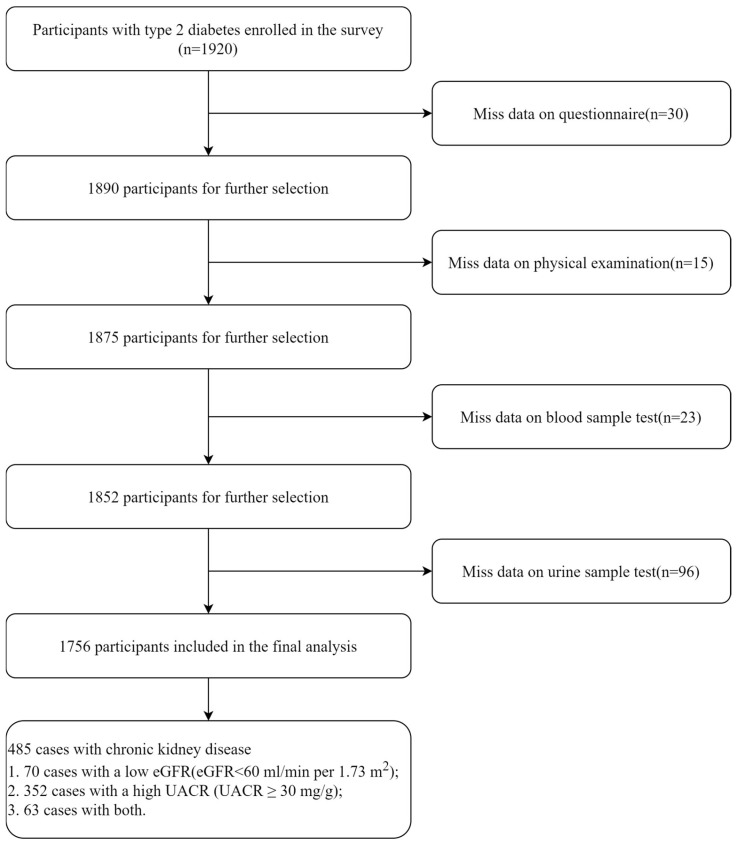
The flowchart illustrating the selection criteria and process for participants included in the final analysis.

A multi-stage random sampling method was employed for participant selection. First, 2 districts and 2 counties within Zhejiang Province were selected. Next, 4 towns were randomly chosen within each district or county. Finally, 120 T2DM individuals were randomly selected from each town, with the selection process stratified by gender and age, resulting in a total of 1920 individuals. Each participant underwent a comprehensive physical examination, provided fasting blood and urine samples, and completed a detailed in-person questionnaire. Participants with missing questionnaire, physical examination, blood sample test, or urine sample test data were excluded from the analysis. After exclusions, a total of 1756 participants were included in the final analysis. The flowchart of the sample selection process is presented in Figure 1.

### 2.2. Data Collection

The data collection process was carried out by qualified professionals affiliated with local centers for disease control and prevention and primary healthcare institutions. These trained individuals conducted structured in-person interviews to methodically gather comprehensive data on participants’ demographic profiles, lifestyle habits, and other relevant variables. Standardized physical examinations were performed by experienced healthcare providers at primary healthcare institutions, which involved accurate measurements of weight, height, waist circumference (WC), and blood pressure (BP). Weight was recorded using an electronic scale (HD-390, TANITA, Tokyo, Japan), while height was measured using a stadiometer (TZG-210, Boyou, Shanghai, China). WC was measured with a standardized waist measuring tape at the midpoint between the lower rib margin and the iliac crest at the end of normal expiration. BP was measured using an electronic BP monitor (HBP-1300, OMRON, Kyoto, Japan), with three consecutive readings taken at one-minute intervals.

Blood samples were obtained from participants after an overnight fast, and first-morning urine specimens were collected to evaluate various biochemical parameters, including fasting plasma glucose (FPG), hemoglobin A1c (HbA1c), triglycerides (TGs), total cholesterol (TC), low-density lipoprotein cholesterol (LDL-C), high-density lipoprotein cholesterol (HDL-C), serum uric acid (SUA), urinary albumin (UAlb), and urinary creatinine (Ucr). Lipid profile analysis (TC, TG, HDL-C, LDL-C), SUA, and Ucr were measured using enzymatic methods, while UAlb was measured using the immunoturbidimetry method on an automated analyzer (Cobas C701, Roche, Basel, Switzerland). FPG levels were determined via a hexokinase assay, and HbA1c quantification was performed using high-performance liquid chromatography on a Hemoglobin Analyzer (D10, Bio-Rad, Berkeley, CA, USA). These methods ensured high precision and reliability in all biochemical measurements.

### 2.3. Definition of the Variables

The primary outcome was CKD in participants with T2DM, characterized by impaired kidney function [estimated glomerular filtration rate(eGFR) < 60 mL/min per 1.73 m^2^] and/or albuminuria (urinary albumin-to-creatinine ratio (UACR) ≥ 30 mg/g) [15]. The eGFR was determined using the CKD-EPI equation [16]. Hypertension was identified as either systolic blood pressure (SBP) ≥ 140 mmHg and/or diastolic blood pressure (DBP) ≥ 90 mmHg, along with a self-reported diagnosis of hypertension by hospitals [17]. An unfavorable lipid profile was characterized by meeting any of the following criteria: TC levels ≥ 6.22 mmol/L, TG levels ≥ 2.26 mmol/L, LDL-C levels ≥ 4.14 mmol/L, or HDL-C levels < 1.04 mmol/L [18]. Abnormal FPG and HbA1c values were defined as readings ≥ 7.0 mmol/L and ≥7.0%, respectively. Educational levels were categorized as secondary school or lower, senior high school, and college or higher. Participants were stratified into age groups: young adults (18–44 years), middle-aged adults (45–59 years), and older adults (≥60 years). Participants were classified as urban or rural residents based on their living areas. Current smoking status was confirmed by daily or occasional cigarette use, and alcohol consumption was recorded if participants reported drinking within the last 30 days.

### 2.4. Definition of the TyG-BRI Index

The *TyG-BRI* index is calculated as the product of the *TyG* index and the *BRI* index. The BRI is computed using the following formula [19]: $BRI=364.2\;-\;365.5\;\times \;\surd (1\;-\;{\left[WC\;(cm)\;/\;2\pi \right]}^{2}/\;{\left[0.5\;\times \;height\;(cm)\right]}^{2})$. The *TyG* index is calculated using the following formula [20]: TyG index=Ln [1/2FPG (mg/dL)×TG (mg/dL)]. Finally, the *TyG-BRI index* is determined by multiplying the *TyG* index by the *BRI* index: TyG−BRI index=TyG index×BRI.

### 2.5. Statistical Analysis

Numerical data were presented as either the mean ± standard deviation (SD) or median (interquartile range), based on the data distribution. For group comparisons, the independent *t*-test was employed for normally distributed data, while the Wilcoxon rank-sum test was used for non-normally distributed data. Categorical variables were described as counts (percentages), and group differences were evaluated using the chi-square (χ^2^) test. To explore factors linked to CKD, a multivariate logistic regression model was utilized. The backward elimination approach was applied to retain the most statistically relevant variables in the final model [21]. The association between the TyG-BRI index and CKD risk was illustrated using restricted cubic splines (RCSs). The predictive capability of the TyG-BRI index for CKD was examined through receiver operating characteristic (ROC) curve analysis [22]. The area under the curve (AUC) was computed to evaluate the diagnostic accuracy of the TyG-BRI index, with the optimal cut-off value determined via the Youden index. Sensitivity and specificity were provided for this cut-off value. A *p*-value of less than 0.05 was considered statistically significant. All statistical analyses were performed using SAS software (SAS Institute Inc. Released 2013. SAS for Windows, Version 9.4. SAS Institute Inc., Cary, NC, USA).

## 3. Results

### 3.1. General Characteristics of Participants

This cross-sectional study enrolled 1920 adults with T2DM, of whom 1756 met the eligibility criteria through provision of complete demographic and clinical datasets (Table 1). Participant characteristics were categorized according to CKD status, revealing comparable gender distribution (male: 49.89%; female: 50.11%) between groups. The participants exhibited a mean age of 57.23 years (SD: 10.15) and an average BMI of 24.76 kg/m^2^ (SD: 3.43). Comparative analysis revealed that individuals with CKD demonstrated significantly elevated anthropometric indices (BMI, BRI), metabolic markers (TyG index, TyG-BRI index), and adverse lipid profiles (higher TC levels, lower HDL-C levels) relative to their non-CKD counterparts. Glycemic dysregulation was more pronounced in the CKD group, as evidenced by higher FPG and HbA1c levels. Furthermore, hypertension was more prevalent, and diabetes duration was longer among CKD patients.

To investigate the association between the TyG-BRI index and CKD, Table 2 displays the baseline characteristics of participants categorized into TyG-BRI quartiles. Key variables spanning demographic, anthropometric, and clinical domains were systematically compared across quartile subgroups. Age distributions demonstrated progressive elevation with ascending TyG-BRI quartiles (*p* = 0.002), while the uppermost quartile exhibited a female-predominant sex ratio and elevated BMI values (both *p* < 0.001). Elevated TyG-BRI quartiles showed graded associations with unfavorable lipid profiles (TC, HDL-C, LDL-C), impaired glycemic regulation (HbA1c, FPG), and increased prevalence of hypertension and CKD, with statistical significance maintained (all *p* < 0.001). However, no significant differences were observed in smoking or drinking behaviors across the quartiles.

### 3.2. Association Between TyG-BRI Index and CKD

To assess potential correlation between the TyG-BRI index and CKD, multivariate logistic regression analyses were performed to compute odds ratios (ORs) and the corresponding 95% confidence intervals (CIs) across quartiles of the index, using the lowest quartile (Q1) as the reference category (Table 3). Initial unadjusted analyses revealed odds ratios of 1.18 (95% CI: 0.85–1.64) for Q2, 2.01 (95% CI: 1.47–2.75) for Q3, and 2.83 (95% CI: 2.08–3.85) for Q4 relative to Q1. Following adjustment for demographic factors (age and sex in Model 2), these estimates showed minimal variation. In the final model (Model 3), which accounted for socioeconomic status (e.g., education), clinical parameters (hypertension, abnormal HbA1c, abnormal FPG, SUA, abnormal TC levels, abnormal HDL-C levels, and diabetes duration), and behavioral factors (drinking alcohol), the adjusted ORs for Q2, Q3, and Q4 were 0.93 (95% CI: 0.65–1.33), 1.33 (95% CI: 0.94–1.88), and 1.57 (95% CI: 1.10–2.25), respectively. A significant dose–response trend across quartiles was evident across all analytical models (all *p* < 0.05), indicating a progressive increase in CKD risk with higher TyG-BRI index levels.

### 3.3. RCS Analysis of the TyG-BRI Index in Relation to CKD Risk and the ROC Curve Assessment

**Figure 2:**
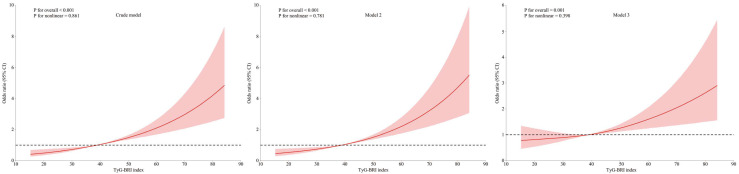
The association between the TyG-BRI index and the risk of chronic kidney disease (CKD) in T2DM patients across different models, allowing for nonlinear effects, with 95% CIs. The solid lines represent the fitted curves between the TyG-BRI index and the risk of CKD. The dotted horizontal lines indicate an odds ratio of 1. The color blocks represent the 95% CIs around the predicted odds ratios. Crude model: unadjusted for any covariates; Model 2: adjusted for age and gender; Model 3: adjusted for age, gender, educational level, hypertension, hemoglobin A1c abnormal, serum uric acid, total cholesterol abnormal, high-density lipoprotein cholesterol abnormal, alcohol drinking, and duration of diabetes. CI, confidence interval; TyG, triglyceride–glucose; BRI, body roundness index.

**Figure 3:**
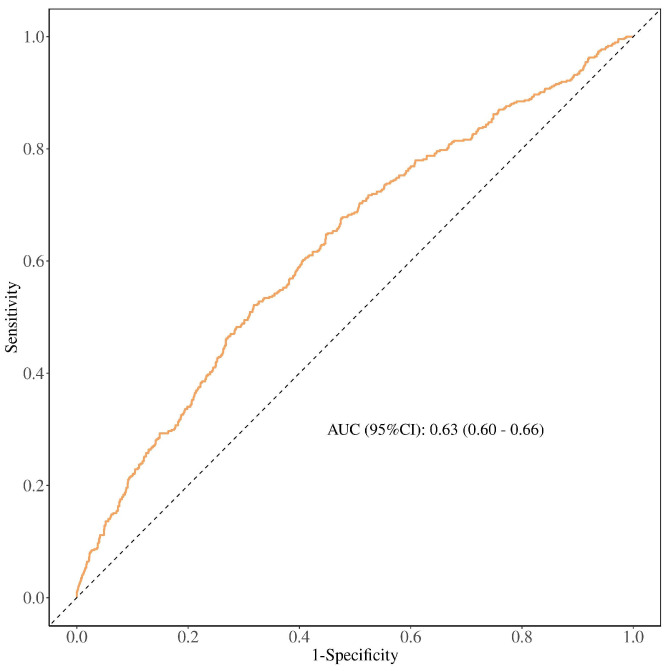
The receiver operating characteristic curve illustrating the diagnostic performance of the TyG-BRI index in predicting chronic kidney disease in patients with type 2 diabetes. AUC, area under curve; CI, confidence interval.

To further investigate the dose–response relationship between the TyG-BRI index and CKD risk, RCS analysis was performed (Figure 2). These spline curves were analyzed across three analytical frameworks: unadjusted, partially adjusted (demographic factors), and fully adjusted models. All models revealed a linear dose–response relationship between the TyG-BRI index and CKD risk (all *p* for nonlinearity > 0.05), with CKD risk escalation observed alongside rising TyG-BRI index levels. The discriminatory capacity of the TyG-BRI index for CKD prediction in T2DM patients was evaluated through ROC curve analysis. This evaluation demonstrated an AUC of 0.626 (95% CI: 0.597–0.656; *p* <0.001). The derived optimal cut-off value was 42.46 (Figure 3), with values exceeding this level demonstrating modest predictive utility. Operational characteristics at this cut-off value included a sensitivity of 68.2% and specificity of 52.2%.

## 4. Discussion

CKD is a serious health issue, particularly among individuals with T2DM [23]. Research indicates that the risk of developing CKD is markedly elevated in T2DM patients compared to their non-diabetic counterparts [24]. According to our findings, CKD prevalence in T2DM patients reached 27.62% in 2018, a figure that significantly exceeds the 8.2% prevalence rate documented in the general Chinese population [15] and the 9.88% rate noted in Zhejiang Province [25]. The increasing prevalence of CKD in individuals with T2DM not only intensifies the burden on affected individuals but also on healthcare systems, further underscoring the importance of early detection and intervention.

This study examined the relationship between the TyG-BRI index and CKD among T2DM patients in Eastern China. The findings indicate that the TyG-BRI index, a combined measure of IR and obesity, was significantly linked to an increased risk of CKD, even after accounting for various potential confounders. A dose–response pattern was observed, with progressively higher TyG-BRI index quartiles associated with an elevated CKD risk. RCS analysis validated a linear association between the TyG-BRI index and CKD risk. Additionally, ROC curve analysis revealed that the TyG-BRI index exhibited moderate accuracy in predicting CKD, with an optimal cut-off value of 42.46.

The observed association between the TyG-BRI index and CKD aligns with the established pathophysiological mechanisms linking IR and obesity to renal damage. IR, a hallmark of T2DM, is closely associated with CKD and plays a critical role in its onset and progression across various stages of the disease [26,27,28]. Hyperinsulinemia, a consequence of IR, promotes sodium retention, renal vasoconstriction, and hypertension, all of which are critical risk factors for CKD [29,30]. Furthermore, IR activates inflammatory pathways and oxidative stress, leading to renal cell damage and fibrosis [31]. Elevated insulin levels also stimulate the renin–angiotensin–aldosterone system (RAAS), contributing to increased glomerular pressure, endothelial injury, and glomerulosclerosis [32]. Triglyceride levels, as reflected in the TyG index, are directly linked to inflammation [33]. Elevated triglycerides promote the secretion of pro-inflammatory cytokines such as interleukin-6 (IL-6) and tumor necrosis factor-alpha (TNF-α) by adipose tissue and other immune cells [34]. This chronic inflammatory state contributes to endothelial dysfunction, oxidative stress, and subsequent renal damage. Screening for inflammation in this context can involve measuring biomarkers such as high-sensitivity C-reactive protein (hs-CRP), IL-6, and TNF-α, which are indicative of systemic inflammation and may serve as early indicators of CKD risk in individuals with elevated TyG index values [34]. Obesity further exacerbates the risk of CKD through multiple interconnected mechanisms, including systemic and renal inflammation, hemodynamic alterations, dyslipidemia, and RAAS activation [35,36,37,38]. Visceral adipose tissue secretes pro-inflammatory cytokines and adipokines, such as leptin and resistin, which not only induce oxidative stress but also promote renal fibrosis and dysfunction [39]. Obesity-related glomerular hyperfiltration, proteinuria, and ectopic lipid deposition in renal tissues result in lipotoxicity and cellular damage, further accelerating CKD progression [40]. Additionally, obesity-related conditions such as obstructive sleep apnea and metabolic syndrome further amplify kidney injury [41]. Considering the inflammatory burden associated with elevated triglycerides, fibrates may represent a potential therapeutic approach. Fibrates are known to lower triglyceride levels by activating peroxisome proliferator-activated receptor-alpha (PPAR-α), which enhances lipid metabolism and reduces the secretion of pro-inflammatory cytokines. Beyond their lipid-lowering effects, fibrates have been shown to exhibit anti-inflammatory and antioxidative properties, potentially mitigating the renal damage associated with chronic inflammation. Clinical trials have demonstrated that fibrates can reduce albuminuria, a marker of renal injury, in patients with T2DM, suggesting a protective effect on renal function. Collectively, these mechanisms drive glomerular and tubular damage, fibrosis, and progressive renal dysfunction, underscoring the complex interplay between IR, obesity, and CKD. In addition to its role in CKD, the TyG index could also have significant implications in predicting cardiovascular events, such as acute coronary syndrome (ACS), particularly in CKD patients [42]. The systemic inflammation and endothelial dysfunction associated with elevated triglyceride levels contribute not only to renal damage but also to an increased risk of atherosclerosis and ACS [43]. The predictive value of the TyG index for ACS in CKD patients warrants further exploration, as it could become a useful biomarker for identifying individuals at high risk of cardiovascular events, thereby enhancing preventive strategies in clinical practice.

Our results align with prior research highlighting the significance of IR and obesity in the progression of kidney dysfunction. For example, a study carried out by Zhao et al. indicates that the higher the TyG index, the more severe the renal vascular damage [44]. Another cohort study involving 11,712 subjects found that the TyG index can predict the occurrence of CKD [45]. A study which enrolled 1432 T2DM patients found that T2DM patients with a higher TyG index had a higher risk of microalbuminuria, and patients in the high tertile of the TyG index at baseline had a greater risk of developing DKD than those in the low tertile [46]. Furthermore, several studies have explored the relationship between the combination of the TyG index and fat distribution indicators in kidney impairment. One such study involving 1080 T2DM patients found that TyG-BMI was associated with renal impairment [47], while another study by Tong Chen et al., focusing on the general Chinese population, suggested that TyG-WHtR had superior diagnostic value for CKD compared to TyG-BMI and TyG-WC [48].

One of the key strengths of this study is the use of the BRI, a novel and more effective indicator of obesity compared to traditional measures such as the BMI or WC. Unlike the BMI, which fails to distinguish between fat and muscle mass, or WC, which does not account for height, the BRI integrates both WC and height to provide a more accurate assessment of body fat distribution and visceral adiposity [49]. This makes the BRI a superior marker for obesity-related health risks, particularly in the context of metabolic and renal complications [50,51]. By integrating the BRI with the TyG index, our study introduces the TyG-BRI index, a composite marker that simultaneously captures IR and obesity-related risk factors. To our knowledge, this is the first study to explore the TyG-BRI index and its association with CKD among T2DM populations in Eastern China.

However, several limitations should be acknowledged. First, the cross-sectional design precludes the establishment of causal relationships. Longitudinal studies are needed to confirm the predictive value of the TyG-BRI index for CKD progression. Second, the study population was limited to T2DM patients in Eastern China, which may limit the generalizability of the findings to other regions or populations. Third, residual confounding cannot be entirely ruled out, despite extensive adjustments.

## 5. Conclusions

This study demonstrates that the TyG-BRI index, a novel marker combining IR and obesity, is significantly associated with CKD risk in T2DM patients, showing a dose–response relationship and moderate predictive value. This study highlights the potential of the TyG-BRI index as a simple and practical method for CKD risk stratification in T2DM patients in clinical settings. These findings contribute to the growing evidence linking IR and obesity to CKD pathogenesis and underscore the importance of integrating metabolic markers into CKD risk stratification.

## Figures and Tables

**Table 1:** The general characteristics of the participants with and without CKD (*n* = 1756).

Characteristics	Overall (*n* = 1756)	Participants Without CKD (*n* = 1271)	Participants with CKD (*n* = 485)	t/χ^2^/z	*p*
Age, mean ± SD, years	57.23 ± 10.15	56.52 ± 9.92	59.09 ± 10.54	−4.77 ^a^	<0.001
Gender, *n* (%)				0.048 ^b^	0.827
Male	876 (49.89)	632 (49.72)	244 (50.31)		
Female	880 (50.11)	639 (50.28)	241 (49.69)		
Educational level, *n* (%)				8.12 ^b^	0.017
Secondary school and lower	1541 (87.76)	1130 (88.90)	411 (84.74)		
Senior high school	171 (9.74)	108 (8.50)	63 (12.99)		
College or above	44 (2.50)	33 (2.60)	11 (2.27)		
Residence, *n* (%)				1.30 ^b^	0.255
Rural	875 (49.83)	644 (50.67)	231 (47.63)		
Urban	881 (50.17)	627 (49.33)	254 (52.37)		
BMI, mean ± SD, kg/m^2^	24.76 ± 3.43	24.51 ± 3.35	25.43 ± 3.56	−5.06 ^a^	<0.001
BRI	4.31 ± 1.23	4.18 ± 1.16	4.65 ± 1.35	−7.31 ^a^	<0.001
Hypertension, *n* (%)	1099 (62.59)	714 (56.18)	385 (79.38)	80.73 ^b^	<0.001
TG, median (IQR), mmol/L	1.60 (1.12–2.42)	1.51 (1.06–2.26)	1.87 (1.30–2.94)	47.47 ^c^	<0.001
TC, mean ± SD, mmol/L	4.65 ± 1.07	4.61 ± 0.97	4.78 ± 1.29	−2.64 ^a^	0.009
HDL-C, mean ± SD, mmol/L	1.25 ± 0.36	1.28 ± 0.35	1.18 ± 0.37	4.96 ^a^	<0.001
LDL-C, mean ± SD, mmol/L	2.73 ± 0.90	2.75 ± 0.85	2.70 ± 1.02	0.98 ^a^	0.325
FPG, mean ± SD, mmol/L	7.94 ± 2.58	7.72 ± 2.31	8.54 ± 3.11	−5.32 ^a^	<0.001
HbA1c, mean ± SD, %	7.27 ± 1.49	7.14 ± 1.40	7.61 ± 1.65	−5.43 ^a^	<0.001
TyG index	9.26 ± 0.73	9.17 ± 0.68	9.49 ± 0.80	−7.89 ^a^	<0.001
TyG-BRI index	40.11 ± 12.63	38.50 ± 11.70	44.30 ± 13.93	−8.13 ^a^	<0.001
SUA, mean ± SD, mmol/L	334.65 ± 94.44	325.89 ± 85.50	357.60 ± 111.48	−5.66 ^a^	<0.001
UAlb, median (IQR), mg/L	17.30 (6.90–43.40)	11.10 (4.95–22.05)	87.10 (41.95–228.00)	705.80 ^c^	<0.001
Ucr, median (IQR), μmol/L	12,735.00 (8799.00–18,085.50)	13,200.00 (9380.50–18,630.50)	11,510.50 (7689.00–16,537.00)	20.04 ^c^	<0.001
Duration of diabetes (years), *n* (%)				23.52 ^b^	<0.001
≤5	836 (47.61)	635 (49.96)	201 (41.44)		
6–10	491 (27.96)	364 (28.64)	127 (26.19)		
11–15	236 (13.44)	148 (11.64)	88 (18.14)		
>15	193 (10.99)	124 (9.76)	69 (14.23)		
Therapies of diabetes, *n* (%)				35.57 ^b^	<0.001
No medication	263 (14.98)	201 (15.81)	62 (12.78)		
Anti-hyperglycemic drugs only	1207 (68.74)	904 (71.13)	303 (62.47)		
Insulin only	87 (4.95)	52 (4.09)	35 (7.22)		
Anti-hyperglycemic drugs and insulin	199 (11.33)	114 (8.97)	85 (17.53)		

^a^ Student’s *t*-test; ^b^ Chi-square test; ^c^ Wilcoxon rank-sum test. Abbreviations: CKD, chronic kidney disease; BMI, body mass index; BRI, body roundness index; TG, triglyceride; TC, total cholesterol; HDL-C, high-density lipoprotein cholesterol; LDL-C, low-density lipoprotein cholesterol; FPG, fasting plasma glucose; TyG, triglyceride–glucose; SUA, serum uric acid; UAlb, urinary albumin; Ucr, urinary creatinine; HbA1c, hemoglobin A1c; SD, standard deviation; IQR, interquartile range.

**Table 2:** General characteristics according to TyG-BRI index quartiles (*n* = 1756).

Variables	Total (*n* = 1756)	Q1 (*n* = 441)	Q2 (*n* = 438)	Q3 (*n* = 439)	Q4 (*n* = 438)	*p*
Age, mean ± SD	57.23 ± 10.15	55.71 ± 10.77	57.33 ± 9.18	58.12 ± 9.85	57.76 ± 10.60	0.002
Gender, *n* (%)						<0.001
Female	880 (50.11)	198 (44.90)	211 (48.17)	211 (48.06)	260 (59.36)	
Male	876 (49.89)	243 (55.10)	227 (51.83)	228 (51.94)	178 (40.64)	
Educational level, *n* (%)						<0.001
Secondary school and lower	1541 (87.76)	355 (80.50)	397 (90.64)	397 (90.43)	392 (89.50)	
Senior high school	171 (9.74)	64 (14.51)	37 (8.45)	32 (7.29)	38 (8.68)	
College or above	44 (2.51)	22 (4.99)	4 (0.91)	10 (2.28)	8 (1.83)	
Residence, *n* (%)						0.054
Rural	875 (49.83)	199 (45.12)	228 (52.05)	213 (48.52)	235 (53.65)	
Urban	881 (50.17)	242 (54.88)	210 (47.95)	226 (51.48)	203 (46.35)	
BMI, mean ± SD	24.76 ± 3.43	21.37 ± 2.01	23.80 ± 1.78	25.47 ± 1.99	28.43 ± 3.09	<0.001
BRI, mean ± SD	4.31 ± 1.23	2.93 ± 0.50	3.88 ± 0.30	4.55 ± 0.38	5.88 ± 0.97	<0.001
TC abnormal, *n* (%)	120 (6.83)	18 (4.08)	19 (4.34)	33 (7.52)	50 (11.42)	<0.001
TG abnormal, *n* (%)	504 (28.70)	32 (7.26)	89 (20.32)	154 (35.08)	229 (52.28)	<0.001
HDL-C abnormal, *n* (%)	506 (28.82)	66 (14.97)	112 (25.57)	153 (34.85)	175 (39.95)	<0.001
LDL-C abnormal, *n* (%)	106 (6.04)	21 (4.76)	21 (4.79)	29 (6.61)	35 (7.99)	0.130
HbA1c abnormal, *n* (%)	859 (48.92)	176 (39.91)	194 (44.29)	215 (48.97)	274 (62.56)	<0.001
FPG abnormal, *n* (%)	989 (56.32)	206 (46.71)	222 (50.68)	262 (59.68)	299 (68.26)	<0.001
Hypertension, *n* (%)	1099 (62.59)	185 (41.95)	259 (59.13)	312 (71.07)	343 (78.31)	<0.001
Smoking, *n* (%)	436 (24.83)	111 (25.17)	122 (27.85)	112 (25.51)	91 (20.78)	0.105
Drinking, *n* (%)	646 (36.79)	162 (36.73)	159 (36.30)	167 (38.04)	158 (36.07)	0.932
CKD, *n* (%)	485 (27.62)	82 (18.59)	93 (21.23)	138 (31.44)	172 (39.27)	<0.001

Abbreviations: CKD, chronic kidney disease; BMI, body mass index; BRI, body roundness index; TG, triglyceride; TC, total cholesterol; HDL-C, high-density lipoprotein cholesterol; LDL-C, low-density lipoprotein cholesterol; FPG, fasting plasma glucose; TyG, triglyceride–glucose; HbA1c, hemoglobin A1c; Q1, first quartile; Q2, second quartile; Q3, third quartile; Q4, fourth quartile.

**Table 3:** The multivariable ORs and 95% CIs for the association of the TyG-BRI index with CKD.

	Crude Model		Model 2		Model 3	
	OR (95% CI)	*p*	OR (95% CI)	*p*	OR (95% CI)	*p*
TyG-BRI index	1.04 (1.03–1.05)	<0.001	1.04 (1.03–1.05)	<0.001	1.02 (1.01–1.03)	<0.001
TyG-BRI index quartile						
Q1	1.00 (ref)		1.00 (ref)		1.00 (ref)	
Q2	1.18 (0.85–1.64)	0.328	1.19 (0.85–1.66)	0.317	0.93 (0.65–1.33)	0.708
Q3	2.01 (1.47–2.75)	<0.001	1.95 (1.42–2.67)	<0.001	1.33 (0.94–1.88)	0.108
Q4	2.83 (2.08–3.85)	<0.001	2.82 (2.07–3.85)	<0.001	1.57 (1.10–2.25)	0.014
*p* for trend		<0.001		<0.001		0.003

Abbreviations: OR, odds ratio; CI, confidence interval; TyG, triglyceride–glucose; BRI, body roundness index; CKD, chronic kidney disease; Q1, first quartile; Q2, second quartile; Q3, third quartile; Q4, fourth quartile; ref, reference. Crude Model: unadjusted for any covariate; Model 2: adjusted for age and gender; Model 3: adjusted for age, gender, educational level, hypertension, hemoglobin A1c abnormal, fasting plasma glucose abnormal, serum uric acid, total cholesterol abnormal, high-density lipoprotein cholesterol abnormal, alcohol drinking, and duration of diabetes.

## Data Availability

The data presented in this study are available on request from the corresponding author (The data are not publicly available due to privacy restrictions).
